# Optogenetic stimulation of complex spatio-temporal activity patterns by acousto-optic light steering probes cerebellar granular layer integrative properties

**DOI:** 10.1038/s41598-018-32017-w

**Published:** 2018-09-13

**Authors:** Oscar Hernandez, Katarzyna Pietrajtis, Benjamin Mathieu, Stéphane Dieudonné

**Affiliations:** 1 Institut de biologie de l’Ecole normale supérieure (IBENS), Ecole normale supérieure, CNRS, INSERM, PSL Université, 46 rue d’Ulm, 75005 Paris, France; 2 Wavefront-engineering Microscopy Group, Neurophotonics Laboratory, CNRS UMR8250, Paris Descartes University, Sorbonne Paris Cité, 45 rue des Saints-Pères, 75270 Paris Cedex 06, France; 3 Present Address: CNC Program, Stanford University, Stanford, California 94305 USA

## Abstract

Optogenetics provides tools to control afferent activity in brain microcircuits. However, this requires optical methods that can evoke asynchronous and coordinated activity within neuronal ensembles in a spatio-temporally precise way. Here we describe a light patterning method, which combines MHz acousto-optic beam steering and adjustable low numerical aperture Gaussian beams, to achieve fast 2D targeting in scattering tissue. Using mossy fiber afferents to the cerebellar cortex as a testbed, we demonstrate single fiber optogenetic stimulation with micron-scale lateral resolution, >100 µm depth-penetration and 0.1 ms spiking precision. Protracted spatio-temporal patterns of light delivered by our illumination system evoked sustained asynchronous mossy fiber activity with excellent repeatability. Combining optical and electrical stimulations, we show that the cerebellar granular layer performs nonlinear integration, whereby sustained mossy fiber activity provides a permissive context for the transmission of salient inputs, enriching combinatorial views on mossy fiber pattern separation.

## Introduction

Optogenetic indicators and actuators are changing the face of modern neurosciences by enabling large-scale, cell-type specific manipulation and recording of neural activity with light^1–3^. Using simple illumination methods, researchers have been able to successfully activate or inhibit large neuronal populations^4–8^ and provide insights about numerous brain functions and behaviors^9^. However, in order to mimic physiological activity patterns with light, sophisticated optical methods that can engage each cell with a specific temporal profile are needed^2,10,11^.

The degree of spatial confinement required for optogenetic stimulations with single-cell precision is preferably achieved with multi-photon excitation^2,12–14^. However, due to low conductivity^15^ and low density of most optogenetic actuators^16^, efficient two-photon optogenetic manipulation requires quasi-simultaneous excitation of optogenetic tools over large areas of the target cells membranes^17,18^. This can be achieved either by quickly scanning a tightly focused beam^19^ or by simultaneous illumination^14,20^. Both approaches have been successfully used for single-cell precision optogenetic stimulation in cultured neurons, brain slices and *in vivo*^19,21–24^ and some of them are capable of imprinting activity in three dimensions with single-cell precision^12,25–27^. However, scanning-based approaches have a low spike precision (on the order of milliseconds or even tens of milliseconds)^19^, due to scanning and dwell times, while parallel-based multiphoton approaches necessitate higher illumination peak powers^28^ and generally create artificial synchrony. Both factors limit the total number of cells that can be made to fire (the largest ensemble of neurons that has been activated within physiological time-scales (<10 ms) has not yet exceeded a few tens^24,29^), as well as the temporal coordination of these cells’ activity, thus restricting the experimenter capacity to mimic physiological activity patterns.

Here we present an ultra-fast single-photon optical method that enables stimulation of large neural ensembles with single-cell lateral accuracy and sub-millisecond spike timing precision. Our system takes advantage of the efficiency of laterally extended light patterns (i.e. low-NA Gaussian beam) and combines it with the remarkable pointing speed of AODs. Contrary to previous AOD-based systems, our approach utilizes only a small optical window at the level of the AOD crystal, enabling unprecedented microsecond-scale pointing speed while preserving sub-micron precision. These unique capabilities, approximately two orders of magnitude faster than systems based on digital micro-mirror devices (DMDs) and three orders of magnitude faster than galvanometric mirrors, are optimal for imprinting asynchronous – and more physiological – neural activity patterns in brain microcircuits using tailored spatio-temporal patterns of light.

We calibrated our system by evoking direct ChR2 current in cerebellar Purkinje cells, whose dendrites acted as a thin planar ChR-expressing layer, and found that ChR2 saturation occurred at low energy. We then demonstrated that sub-millisecond light pulses can stimulate cerebellar mossy fibers (MFs) expressing ChR2 with single-terminal and 100 µs precision up to a depth of 100 µm. Using an optimized algorithm for the delivery of spatio-temporal illumination patterns, we evoke sustained and repeatable asynchronous patterns of MF population activity. Pairing these patterns with minimal electrical stimulations allowed us to probe the mechanisms for sensory-motor coding by granule cells in the cerebellar cortex. We found that asynchronous MFs activity could provide the context in which salient stimuli are transferred through the granular layer and demonstrated that this nonlinear transfer is under the inhibitory control of Golgi interneurons.

## Results

### General system design

**Figure 1:**
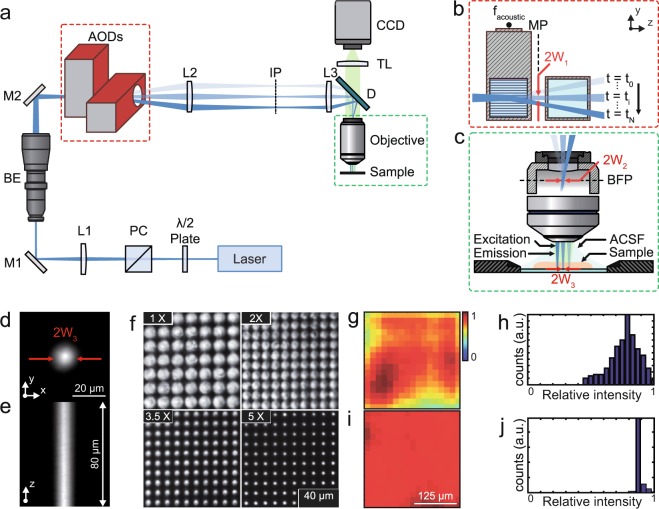
Schematic of the optical setup and characterization. (**a**) A half-wave (λ/2) plate and a polarizer cube (PC) modulate the laser power and assure linear polarization necessary for the correct functioning of the AODs. The lens L1 focuses the beam on the AOD medial plane (MP) after passing through a variable beam expander (BE) that controls the beam waist. The beam is then relayed onto the objective rear aperture by the lenses L2 and L3. The resulting magnification underfills the objective back aperture producing a large excitation volume at the sample plane. Emitted fluorescence is collected through the same objective and imaged on a CCD camera through a tube lens (TL). (**b**) The beam is focused at the AOD medial plane (MP) and laterally deflected according to the acoustic frequency (f_acoustic_) driven into the AODs. (**c**) The MP is imaged at the objective back focal plane (BFP) so all beams exiting the objective are parallel to the optical axis. (**d**,**e**) Lateral and axial fluorescence intensity distribution at the sample, respectively. (**f**) Spot size at the sample plane corresponding to different magnification values of the beam expander. (**g**) The diffraction power depends in a nonlinear way on the deflection angle. This leads to non-homogeneous power distribution. (**h**) Histogram of the power distribution. (**i**) The power distribution was characterized and dynamically equalized by power modulation of the acoustic signal. (**j**) Histogram of the power distribution after equalization. Abbreviations: TL: tube lens; ACSF: artificial cerebro-spinal fluid; W: beam waist; IP: intermediate plane.

We designed an optical system to achieve fast and efficient one-photon (1P) optogenetic stimulation in live tissue by combining low-numerical aperture (NA) Gaussian beam illumination and fast acousto-optic steering. Figure 1a presents the schematic of the system, where a 473 nm continuous wave diode-pumped solid-state laser (LRS 0473-00100-03, Laserglow Technologies) was used as a 1P light source for ChR2 excitation. The beam was focused on the medial plane of a dual-axis shear-mode tellurium dioxide (TeO_2_) AOD scanner (Fig. 1b) (DTSXY-250-473, AA-Optoelectronic) using a lens (L1) and a beam expander. The AOD medial plane was then conjugated to the back aperture of a 40x objective (Fig. 1c) (40x LUMPlanFl/IR, 0.8 NA; Olympus) by a 4-f system, thus allowing parallel scanning motion of the beam at the output of the objective. The magnification of the 4-f system was chosen to be close to ×0.5, to achieve a theoretical scanning field at the sample of 324 × 324 µm^2^, given a total scanning angle (Δ*θ*) of 36 mrad for each axis of the AOD crystals.

In our system, the size of the beam waist at the conjugated AOD medial plane and objective pupil plane defines the effective objective NA and thus the beam waist diameter at the sample. An example of the axial propagation at the sample plane of a low-NA Gaussian beam (10 µm FWHM; 17 µm diameter at e^−2^) is shown in Fig. 1d,e. Waist tuning at the objective back aperture (W_2_) with constant scanning field was obtained by placing a variable beam expander (2x–5x, BE02-05-A, Thorlabs) before the AODs to control the waist W_1_ (Fig. 1b). We designed the system to produce beam waists at the sample plane that ranged from 2 µm to 10 µm in diameter (FWHM; Fig. 1f). Thus, the number of resolved beams at the focal plane was given by the AOD resolution formula considering the angular divergence Div_0_ of the Gaussian beam of diameter 2W_1_ at the AODs plane (N = *Δθ*/Div_0_). In our conditions 2W_1_ ranged from 332 µm to 1660 µm, which translates into switching times of 0.5 µs for the widest beam at the focal plane to 2.5 µs for the thinnest one.

Although AODs in acoustically rotated phase matching configurations take full advantage of the large figure of merit of TeO_2_^30^, and provide a large bandwidth without degeneration mode^31^, the acousto-optic bandshape is not completely uniform^31–34^. In our system, the transmitted optical power variation in the field of view reached up to 25% (Fig. 1g,h). To equalize it we scaled the acoustic power according to each scanning angle/acoustic frequency (Fig. 1i,j)^35^. To this end, we measured the laser diffraction efficiency as a function of the acoustic power (Suppl. Fig. 1a) and the deflection angle (Suppl. Fig. 1b). Then, we designed a simple algorithm (Suppl. Fig. 1a) to adjust the acoustic power dynamically, thus decreasing variations of laser intensity down to ~2.6% (Suppl. Fig. 1c,d).

### Low numerical-aperture Gaussian beams reduce the impact of scattering on the spatial contrast of one photon stimulation in depth

**Figure 2:**
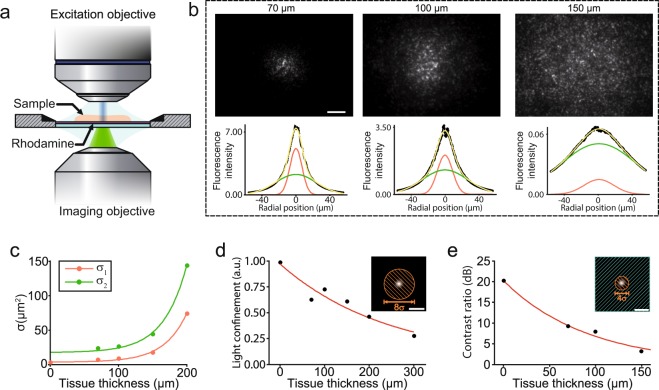
Characterization of low-NA Gaussian beam propagation through brain slices. (**a**) Two objectives positioned in opposed direction and focused on the sample plane to form a double microscope. This configuration enables high resolution imaging of the speckle patterns formed after beam propagation. In this case, a low-NA Gaussian beam exits the upper objective and propagates through a brain slice of a certain thickness towards a thin fluorescent layer placed beneath the sample. The emitted fluorescence is collected by the bottom objective and imaged on a CCD camera. (**b**) Upper panel images represent the spatial distribution of light on the rhodamine layer after propagating through brain slices of 50, 100 and 150 µm thicknesses, respectively. The graphs below represent a radial distribution of light. Dotted black line represents the experimental data fitted with curve (yellow line) resulting from the addition of two Gaussian curves (red and green lines) with different amplitude and standard deviation. The variation of the relative amplitude and standard deviation of the two Gaussians with sample thickness suggest that the two components are mostly related to ballistic or single scattering photons (red line) and multiple scattering photons (green line). Scale bar; 10 µm. (**c**) Degradation of the lateral precision with depth seen as the variation on the standard deviation of the Gaussian curves. (**d**) Normalized integrated fluorescence intensity within a 8*σ*_1_-dimenter disk for different tissue thicknesses. (**e**) Ratio between integrated fluorescence intensity within a 4*σ*_1_-diamenter disk (orange area) and the integrated fluorescence intensity on the outer area (green area) for different tissue thicknesses.

Light scattering degrades the beam profile as it penetrates deeper in brain tissue^36–38^. For visible light, the scattering coefficient is at least one or two orders of magnitude higher than the absorption coefficient^36^ limiting the capability to achieve sub-micron precision at depths larger than 100 µm^39^. At the particular wavelength used in this paper the contribution of Mie and Rayleigh scattering are comparable^40^ yielding to milder detrimental effects, as low-NA Gaussian beams are axially elongated and Mie scattering produces mostly forward-peaked scattering light distributions^40–42^ with an anisotropy parameter close to g ≈ 0.9^36,37^. To assess the impact of scattering, a low-NA Gaussian beam was propagated through acute brain slices of different thickness (Fig. 2a) and a thin fluorescent layer was placed under the brain tissue to provide a cross-section of the beam exiting the tissue. The fluorescence photons emitted were collected through a second objective located under the sample and imaged onto a CCD camera.

Brain tissue scatters coherent light in a variety of directions producing speckled illumination by mutual interference^43^. When these speckled patterns are averaged, two components can be distinguished and fitted by two Gaussians^44^ that primarily correspond to ballistic and scattered light, respectively. Light distribution at different depths can be estimated by the relative amplitudes (*A*_1_ and *A*_2_) of the two components and by their corresponding standard deviations (*σ*_1_ and *σ*_2_) (Fig. 2b), which increase exponentially with depth (Fig. 2c). Interestingly, the diameter of the narrower Gaussian component only increased from 8 µm to 21 µm in the first 100 µm. The fraction of intensity confined within diameter (8*σ*_1_) of the illumination beam decreased exponentially with depth (Fig. 2d) with a space constant of 140 µm, similarly to previously reported values^36,45,46^ and simulations^47^. The contrast ratio between the integrated intensity in the central beam (4*σ*_1_ disk) and the rest of the scattered light remained as high as 9.3 dB at 70 µm and 8.0 dB at 100 µm depth (Fig. 2e) proving the potential of our low-NA Gaussian beams to laterally confine light at depths ≥ 100 µm inside brain tissue.

### Optogenetic stimulation model and calibration

**Figure 3:**
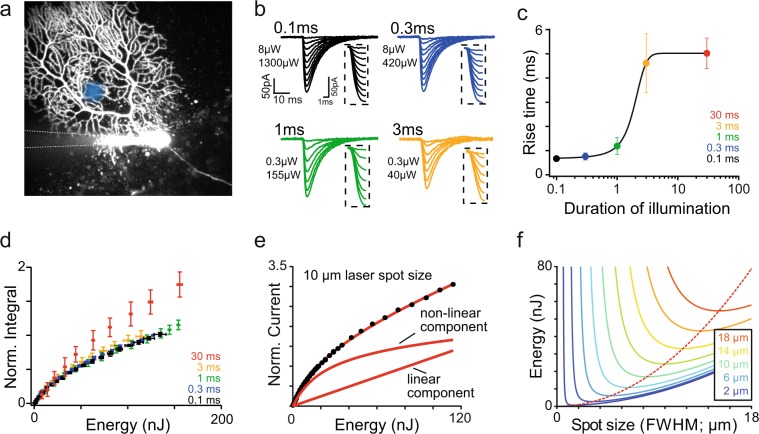
Calibration of ChR2 activation by Gaussian beams. (**a**) Schematic for experimental configuration with two-photon image of Purkinje cell filled with morphological dye (Alexa 594, 15 µM) via recording pipette in L7-ChR2-YFP mouse expressing ChR2 with schematic position of laser single beam (blue circle). (**b**) Average traces of whole-cell currents recorded from the soma of the Purkinje cell in response to single beam (~10 µm) stimulation of the dendritic tree with different duration of illumination and at increasing light intensity. Each trace is the average of 10 stimulations. Values in µW indicate the smallest (top) and highest (bottom) power used in the power increment protocol. Activation of each average for a given duration of illumination is enlarged in inset on the right side of each trace. **c**. Dependence of the rise time of the light-evoked currents (in ms) on the duration of illumination (presented in logarithmic scale). Stimulation duration averages are colour coded (black: 0.1 ms, blue: 0.3 ms, green: 1 ms, orange: 3 ms and red: 30 ms). Data points represent the mean ± s.d. (n = 6 cells). (**d**) Integral of the light-evoked currents as a function of energy. For pulses of 3 ms and shorter, charge carried by delivering the same amount of energy will remain the same – activation curve is similar regardless of duration of illumination. Each response curve corresponds to the traces shown in b. Data points are mean ± s.d. (n = 8 cells). (**e**) Recruitment curve for ChR2 currents recorded at the Purkinje cell soma for single beam stimulation of 0.1 ms. The curve is well fitted by our ChR2 current recruitment model (red curve) (see Methods). Below two components (linear and non-linear) are presented. Saturation energy for ChR2 obtained from this model is 0.067 nJ µm^−2^. (**f**) Energy required to achieve ChR2 saturation within a circular area for a given size of the illumination spot. The different solid color curves indicate the size of the saturated region and the red-dash line the illumination spot size that provides saturation in a given region with the lowest amount of energy possible.

High laser intensities yield faster ChR2 activation but can deteriorate the spatial stimulation profile when reaching the ChR2 saturation level. To determine the best intensity tradeoff in realistic experimental conditions, we used the dense and planar dendritic tree of ChR2-expressing Purkinje cells as a thin photosensitive layer. Optogenetic currents were recorded at the soma in response to the illumination of Purkinje cells dendrites located at 50 µm depth, in acute slices from L7-ChR2 transgenic mice^48^ (Fig. 3a). Brief light pulses (0.1 ms–30 ms duration) evoked transient currents, with a decay time constant of about 8 ms (7.79 ± 1.06 ms for 30 ms, 7.98 ± 0.78 ms for 1 ms and 8.3 ± 1.30 ms for 0.1 ms), in agreement with the measured deactivation kinetic of ChR2 (Fig. 3b). At constant light pulse energy, current activation time constants (10–90%) decreased with pulse duration from 5.02 ± 0.63 ms for 30 ms pulses to 0.67 ± 0.1 ms for 0.1 ms pulses (Fig. 3c), while preserving the charge of the optogenetic current generated (Fig. 3d). Efficient stimulation can thus be implemented by very short light pulses, allowing the use of the full dynamics of our AOD system. For pulses of 30 ms the charge was increased by 26% (n = 7; compared to 1 ms pulses) (Fig. 3d), suggesting that multiple activations of the same ChR2 molecules can occur if the illumination time exceeds the deactivation time.

The recruitment curve of ChR2 currents was clearly sub-linear at energies above 20 nJ, but never fully saturated (Fig. 3d). To find the optimal stimulation level that yield activation without compromising spatial resolution, we developed a mathematical model based on stochastic ChR2 activation by low-NA beams (Methods). In our model, the total ChR2 conductance is calculated by spatial integration of the laser intensity, thus considering a gradual saturation of ChR2 activations near the beam center due to high radiant exposure. This yielded to a non-linear component - the sum of an exponential integral function and a logarithmic function. The second component of the model is linear and accounts for low-intensity scattered light activation around the target that do not saturate ChR2 (Fig. 3e). The complete model is described by Eq. 1. The ChR2 current density (d_ChR2_), the characteristic radiant exposure saturation constant for ChR2 molecules (RE_0_) and the fraction of current activated by scattered light (α_s_) are the only free parameters:

1 $${C}_{ChR2}=2\pi {\sigma }_{r}^{2}{d}_{ChR2}\sum _{n=1}^{\infty }\,\frac{{(-1)}^{n+1}{(\frac{Pd}{2\pi {\sigma }_{r}^{2}R{E}_{0}})}^{n}}{nn!}+{\alpha }_{s}Pd$$

with *P* being the laser power, *d* the pulse duration and 2*σ*_r_ the beam waist. Eq. 1 was used to fit the experimental data (MSE < 10^−4^) for various pulse durations (Fig. 3c) yielding the following parameters RE_0_ of 79 ± 18 pJ μm^−2^ (d_ChR2_ = 1.6 ± 0.25 fA μm^−2^, α_s_ = 3.4 ± 0.38 fA nJ^−1^).

Lastly, we calculated the energy required to saturate ChR2 in a circular area of a given diameter as a function of the beam waist (Fig. 3f). Each curve of this family shows a global minimum, yielding the optimal beam waist diameter to saturate ChR2 in a given area with the minimum amount of energy possible, thus maximizing the spatial stimulation precision.

### Optogenetic stimulation of single neurons with sub-milisecond and micron precision

**Figure 4:**
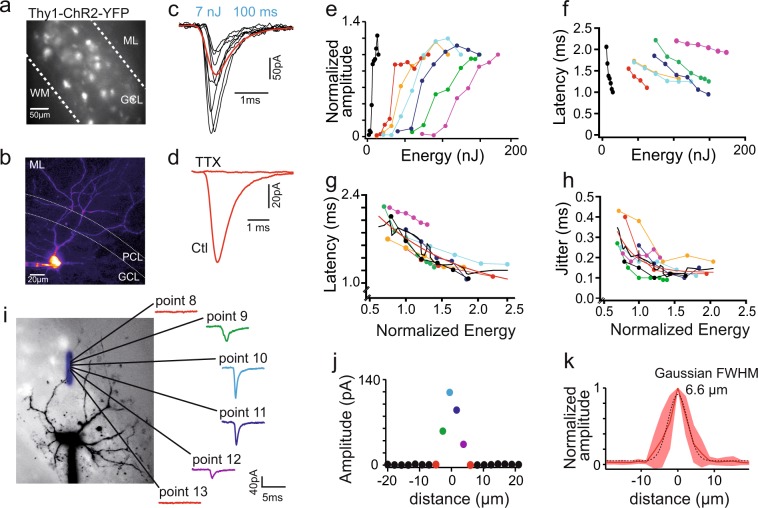
Optogenetic stimulation of single mossy fibers with submilisecond precision and spatial precision of low-NA Gaussian beam stimulation. (**a**) Fluorescence image of cerebellar slice from the vermis of a Thy1-ChR2-YFP mouse that expresses ChR2 and YFP in a subset of mossy fibers. (**b**) Two-photon image of the Golgi cell filled with Alexa 594 (15 µM) via the recording pipette; GCL: granule cell layer, PCL: Purkinje cell layer; ML: molecular layer, WM: white matter. (**c**) Top panel: average EPSC (in red) evoked by the 100 µs stimulation with examples of single events (in black). (**d**) Light evoked events are action-potential dependent since the response disappears in the presence of TTX (200 nM). (**e**) Energy response curve recorded in Golgi cells (n = 7). (**f**) EPSCs latencies (in ms) as a function of energy for each cell (color code the same as in e); latencies measured on averages from 100 events, EPSCs delay decreased with increasing laser power. (**g**) Light evoked events latencies (as in f) as a function of energy which was normalized to the half activation value, extracted from sigmoidal fit applied to the recruitment curves in e; values of the fit: start 2.06 ms, tau: 1.197, base: 0.78 ms. Color code as in e and f. (**h**) Jitter, defined as the standard deviation of the latency, as a function of energy, normalized as described in g. Values of the fit: start 0.327 ms, tau: 0.273, base: 0.126 ms. Color code the same as in e–g. (**i**) two-photon image of the Golgi cell recorded during spatial resolution experiment with the drawing of the accurate position of the stimulated points (superimposed in blue). The center of each point is marked with a black dot. On the right, examples of the evoked EPSCs in one cell showing no response (point 8 and point 13), response evoked at the edge of the mossy fiber (before and after the hotspot, point 9 and 12, respectively) and hotspot stimulation in point 10 and 11. Note decreased latency of the events at the hotspot in comparison to the spot located at the edge of the fiber. (**j**) Graph showing the amplitude of the evoked EPSCs as a function of distance for the example cell in c; colors of the spots on the graph correspond to the points in a. **k**. Summary graph showing the relative amplitude (normalized to the maximal amplitude for each cell) of the evoked events as a function of distance, zeroed at the hotspot (maximal evoked amplitude). Shaded area around the average (solid red line) represents ± s.d.; n = 8.

The efficacy of our system at eliciting spikes in neurons was tested on acute brain slices from Thy1-ChR2-YFP transgenic animals (line 18^49^), which express ChR2 in a population of cerebellar MFs (Fig. 4a). MFs form giant varicosities or rosettes (Fig. 4a) that constitute the only excitatory afferents to the granular layer of the cerebellar cortex, where they contact both granule cells and the local inhibitory interneurons, the Golgi cells. MFs, identified by their YFP expression, were stimulated by 100 µs light pulses using 9 µm FWHM beams, while performing whole-cell recordings from Golgi cells (Fig. 4b). Excitatory postsynaptic currents (EPSCs) were evoked at some stimulation loci (Fig. 4c). These EPSCs were fully blocked by TTX (200 nM; p = 0.01; n = 4; t-test) (Fig. 4d) indicating that vesicular release was triggered by regenerative sodium events.

Recruitment curves were performed on these isolated mossy fibers EPSCs (Fig. 4e). All recruitment curves showed a clear threshold and monotonic increase to a plateau value (Suppl. Fig. 2a–c). The half recruitment intensity, estimated from sigmoidal fits, varied widely between stimulation sites (61.9 ± 40.6 nJ, n = 7), ranging from 6.01 nJ, close the expected ChR2 saturation level (5.03 nJ), to 125.8 nJ. These higher threshold energies, together with shallower recruitment curves, most likely reflect the stimulation of deeper targets by scattered beams of degraded peak intensity. Variable levels of ChR2 expression in some fibers may also participate to the large range of threshold intensities.

Around threshold, the failure probability decreased sharply with increasing light intensity and stabilized to 0.15 ± 0.13, n = 7 (Suppl. Fig. 2b). Surprisingly, stable non-failure amplitude was not achieved at threshold, but was preceded by a regime under which small events (43.52 ± 28.11 pA, n = 7 fibers) prevailed (Suppl. Fig. 2c), which were similar in amplitude to miniature EPSCs^50^. The long latency (7.20 ± 0.31 ms) and large jitter (Suppl. Fig. 2a) of these small MF EPSCs suggests that slow MF depolarization at threshold may lead to reduced calcium influx and single vesicle release. However, stimulation latency (1.37 ± 0.32 ms, p = 0.015, n = 7) (Fig. 4f,g) and trial-to-trial jitter (0.14 ± 0.05 ms, n = 7; Fig. 4h) decreased dramatically at higher stimulation intensities and the amplitude of the non-failure light-evoked unitary events (86–163 pA, median 106.5 pA, n = 36; Suppl. Fig. 2b) was comparable to the amplitude of EPSCs evoked by electrical stimulations^51^.

We assessed the spatial resolution of the optical stimulation by moving the beam away from a stimulation hotspot along a line in steps of 2.16 µm while recording the mean synaptic response at each site (Fig. 4i). EPSCs could not be evoked at distances larger than 6.5 µm from the central responsive point (Fig. 4j,k). Overall, the spatial response profile could be fitted by a Gaussian function with a FWHM of 6.6 µm, similar to the diameter of the stimulation beam (9 µm) (Fig. 4k). These experiments confirm that micron-scale optogenetic stimulation precision can be attained in scattering media when using low-NA Gaussian beams. Overall this set of data indicates that our system has the unique capacity to evoke spikes with single-cell and 0.1 ms precision up to 100–150 µm in brain tissue and at 10 kHz rate.

### Optogenetic emulation of sensory-motor activity

The stimulation capabilities of our system are ideal to evoke sustained multi-cell physiological activity patterns. However, in a multi-cell scenario, the spatio-temporal sequencing of the stimulation pattern plays a critical role due to the kinetics and desensitization of optogenetic actuators^4^. To address this issue, we developed and tested an algorithm that generates optimal spatio-temporal stimulation patterns by maximizing light coverage while minimizing cross-talk between neighboring targets (Suppl. Fig. 3). The algorithm first discretizes the stimulation area into a finite number of equidistant points that will be later illuminated by sub-millisecond light pulses. Each point is accessed only once during the sequence to ensure uniform illumination power within the FOV. Finally, the distance between consecutive illumination points in the sequence is maximized by using a time-weighted distance function that sets the illumination sequence order (see Methods).

**Figure 5:**
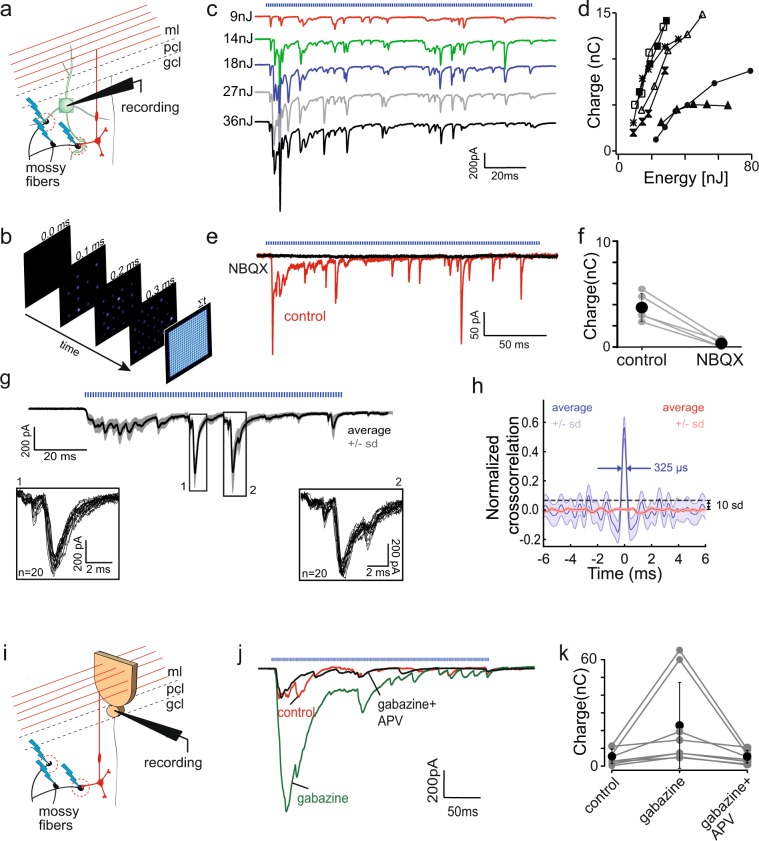
Optogenetic emulation of sensory-motor activity. (**a**) Cartoon showing experimental configuration. Golgi cells in the Thy1-ChR2-YFP vermal slices were recorded in the whole-cell, voltage clamp configuration; optical, asynchronous stimulation, governed by our optimized spatiotemporal light patterns (see Methods), was applied to the field of view surrounding the recorded cell. Blue lightings represent light stimulation. (**b**) Cartoon explaining scanning mode; currently scanning point is represented by the spot with the red filling and fainter points show previous stimulation placement. The last square (farthest right) shows the grid of points covering the whole FOV. (**c**) Recruitment of currents evoked by the asynchronous optogenetic stimulation for 145 ms in one cell (blue trace represents the onset and duration of the stimulation). Energy of each stimulation indicated on the left of each trace. Colors are used only for better visualization of single traces. (**d**) Summary of the charge carried during light stimulated pattern recorded in Golgi cells as a function of energy (n = 7); markers represent different cells. (**e**) Evoked responses are blocked by NBQX (2 µM), an AMPA receptor blocker. Example of average trace in control (red trace) and after application of NBQX (black trace), showing disappearance of the light evoked events. (**f**) Summary of charge carried during light stimulation in control and with the presence of NBQX; black dots represent the average with error bars of ±s.d., grey traces represents the results for single cell. (**g**) Evoked responses are reliable across many trials with the same energy. The example trace showing the average of 50 traces, with (very narrow) shaded area representing ±s.d. Blue pattern above the traces represents duration of optical stimulation. Each point was stimulated for 0.9 ms with a gap of 100 µs before the stimulation of the next. Box 1 and 2 below show the enlargement of the events boxed on the average. Note high reliability and repeatability in light evoked events (boxed areas show overlay of 20 traces). (**h**) Normalized cross-correlation of the first derivative of 20 traces (blue line) and normalized cross-correlation of the first derivative of the same traces after splitting each trace in blocks of 100 samples and randomizing their position (red line) (n = 4). (**i**) Schematic drawing showing experimental configuration. GCL output was monitored by recordings from Purkinje cells in Thy1-ChR2-YFP and asynchronous pattern of optical stimulation of mossy fibers was applied (blue lightning). (**j**) Example traces of light evoke responses recorded in Purkinje cells in control conditions (red), after gabazine application (green) and after further APV application (black). (**k**) Summary of the average charge transfer of the full optical response recorded from Purkinje cells in three pharmacological conditions. Black dots represents averages, error bars are ±s.d., grey circles represent single cells.

We recorded from Golgi cells in Thy1-ChR2-YFP animals and applied the optimized spatio-temporal patterns of light stimulation within a user-defined area restricted to the granular layer (Fig. 5a). To facilitate MF activation, MF excitability was increased by addition of low concentrations of 4-AP (10 µm). At this concentration, 4-AP has been shown to exert minimal effect on the transmission of electrically evoked MF activity to Purkinje cells through the granular layer^52^. Each point was illuminated for 900 µs before the beam was redirected to another point, leaving 100 µs gap between two stimulations (Fig. 5b). The total duration of illumination sequence depended on the number of stimulated points. We found that our spatio-temporal patterns of optogenetic stimulation of ChR2-expressing mossy fibers were able to evoke long (100–200 ms) stretches of sustained excitatory synaptic activity recorded in cerebellar Golgi cells (Fig. 5c). The total synaptic charge transfer increased with pulse energy, through gradual recruitment of MFs (Fig. 5c,d). Application of NBQX (2 µM) blocked 91% of this synaptic charge transfer (Fig. 5e,f) (3.73 ± 1.35 nC in control to 0.335 ± 0.35 nC in NBQX, n = 5) consistent with the prevalence of AMPA receptors at the MF synapse onto Golgi cells^50,51^. Salient large EPSCs, characteristic of some MF inputs, were reliably evoked at particular time-points of each stimulation trial (Fig. 5g, inset 1 and 2). To better assess the repeatability of the onset time of all EPSCs, we took the first derivatives of the original traces, which highlight the fast rising-phase of synaptic events, and performed a cross-correlogram between trials. The normalized cross-correlogram showed a large central peak (Peak signal-to-noise ratio: 29.34 dB ± 1.36) with a FWHM of 295.98 ± 22.2 μs (Fig. 5h), indicative of the sub-millisecond timing precision of the stimulation.

We next investigated whether light evoked MF activity was strong enough to be relayed by granule cells to Purkinje cells. Patterned optogenetic stimulation indeed evoked a barrage of EPSCs in Purkinje cells (Fig. 5i,j). Block of NMDA receptors by APV (50 µM) decreased the response recorded in Purkinje cell by 41% (Suppl. Fig. 4b) from 4.2 ± 3.5 nC to 2.48 ± 1.85 nC (n = 5) (Suppl. Fig. 4c), confirming the contribution of NMDA receptors to MF input integration by granule cells^53,54^. Bath applied gabazine (5 µM), a GABA_A_ receptor antagonist, increased the control response by 351% (from 5.09 ± 4.3 nC to 22.96 ± 25.04 nC in gabazine, p = 0.0078, n = 8) (Fig. 5j,k) confirming the crucial role of Golgi cell inhibition on granular layer information processing. This effect was reversed by application of APV (4.92 ± 3.7 nC, p = 0.74, n = 8) (Fig. 5j,k).

Overall, this set of data constitutes the first evidence that asynchronous patterns of optogenetic stimulation can evoke sustained afferent activity, able to drive a brain microcircuit.

### Contextual gating of salient mossy fiber inputs by ongoing optogenetic stimulation

Finally, we tested whether optogenetically evoked mossy fiber activity could serve as a context for salient, synchronous input, evoked electrically, providing experimental support for nonlinear processing in the granular layer.

**Figure 6:**
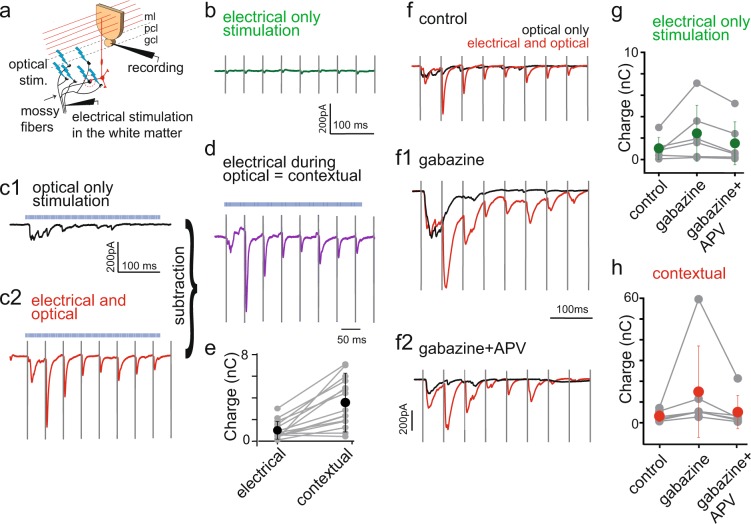
Contextual gating of salient mossy fiber inputs by ongoing optogenetic stimulation. (**a**) Schematic drawing explaining experimental pairing protocol. Cerebellar cortex output activity was measured by recording Purkinje cells in whole-cell configuration. Minimal electrical stimulation of mossy fibers was applied by placing stimulation electrode few hundreds of µm away from the recorded cell body and applying 1 ms pulse at 20 Hz (50 ms interpulse interval) for 4 s. This input was superimposed with the asynchronous stimulation evoked optically (represented by blue lightings), similarly as in previous experiments (Fig. 5). (**b**) Examples of traces from a single cell showing response recorded in Purkinje cell to electrical only stimulation. (**c**) Examples of traces from a single cell showing response recorded in Purkinje cell to (**c1**) optical only stimulation, (**c2**) paired electrical and optical stimulation. (**d**) Trace presenting net electrical stimulation in the context of optical stimulation (contextual charge transfer), calculated by subtracting optical only stimulation from the paired stimulation. (**e**) Summary of the contextual charge transfer (calculated as in d) during the whole period of paired electrical and optical recording; blue dots represent averages ± s.d., grey pairs of points represent single cells (n = 15). (**f**) Contextual gating in the granular layer is under the control of Golgi cell. Examples of traces from a single cell showing response recorded in Purkinje cell to optical only (black trace) or paired electrical and optical stimulation (red trace) in (**f**) control condition, (**f1**) after gabazine and (**f2**) gabazine and APV bath-application (same cell as in c1-c2). (**g**) Summary of average charge transfer during the electrical only stimulation in three pharmacological conditions: control, bath applied gabazine and bath applied gabazine and APV; error bars represents ±s.d. (n = 6). (**h**) Summary of the contextual charge transfer (calculated as for d and e) in three pharmacological conditions (n = 6). Vertical lines in b, c1-c2,d and f-f2 are electrical stimulation artefacts to better visualize the timing of the evoked responses; horizontal, blue dashed line represents optical stimulation.

We placed a monopolar stimulation electrode a few hundreds of micrometers away from the recorded Purkinje cell, in the white matter in order to evoke minimal electrical stimulation of MFs (Fig. 6a). We applied 1 ms square pulses at 20 Hz for 4 s. The sequence was repeated every 10 s. This stimulation will be further referred to as electrical-only stimulation. When presented alone, electrical-only stimulation was filtered in the granule cell layer and little or no response was recorded in Purkinje cell (0.96 ± 0.8 nC, n = 15, Fig. 6b,e). Optical-only asynchronous optogenetic stimulation of the MFs in the granular layer below the recorded Purkinje cell (as described for Fig. 5) evoked a small, sustained response (Fig. 6c1). However, when the two inputs were paired together (optical stimulation started 2 s after the onset of the electrical stimulation) we observed a large potentiation of the electric stimulation-evoked synaptic currents recorded in Purkinje cells (up to a few hundreds of pA; Fig. 6c2). This potentiation was far superior to the predicted linear summation and potentiated events were time locked to the electrical only stimulation (Fig. 6c2). The net synaptic charge evoked by electrical stimulation during the contextual optogenetic stimulation was calculated by subtraction (Fig. 6d) and revealed a nearly 3 times potentiation compared to electrical-only stimulation (Fig. 6e) (3.55 ± 2.26 nC during paired stimulation vs 0.96 ± 0.8 nC for electrical only, n = 15, p = 0.0078, Wilcoxon test). Supra-linear summation occurred within 50 ms of optogenetic stimulation. It persisted for higher electrical stimulations, indicating that it is not just a threshold effect. These results suggest that ongoing asynchronous mossy fiber activity can provide a facilitating context for the transmission of salient or synchronous input, as recently suggested^55^.

Lastly, we studied the impact of GABAergic inhibition and slow NMDA integration on this non-linear information transfer in the granular layer. Bath application of gabazine (5 µM) increased the average charge transfer during paired stimulations by 211% (Fig. 6f-1, red line) (from 7.8 ± 5.9 nC in control to 24.3 ± 29.3 nC with gabazine, p = 0.03, n = 6, Wilcoxon test), which came back to near-control values upon APV application (Fig. 6f2) (10.45 ± 10.7 nC, p = 0.56, n = 6, Wilcoxon test). Gabazine application also increased the charge transfer of the optical only stimulation in 6 out of 7 cells by 265.5 ± 209.6% (2.1 ± 3.2 nC in control vs 8.5 ± 9.2 nC with gabazine) (Fig. 6f–1; black trace) and electrical only by 167 ± 117% (Fig. 6g; 1.1 ± 0.95 nC in control vs 2.6 ± 2.4 nC) which came back to the control level after application of APV (1.5 ± 2.0 nC, p = 0.62, Wilcoxon test). Strikingly, the supra-linear contextual charge transfer evoked by pairing electrical and optical stimulation (Fig. 6h) was potentiated by gabazine more strongly than individual stimulations alone from 3.2 ± 2.5 nC in control to 14.9 ± 22.04 nC, and decreased back to 5.1 ± 8.015 nC upon application of APV.

The excess charge transfer of paired events induced by gabazine was partly mediated by a lengthening of their rise and decay times (Suppl. Fig. 5) (rise time 1.0 ± 0.28 ms and decay 5.7 ± 1.5 ms in control to 1.95 ± 0.73 and 15.2 ± 5 ms in gabazine, respectively). Suppression of the feed-forward inhibition exerted by molecular layer interneurons on Purkinje cell by gabazine could explain this effect. Indeed, disynaptic shunting inhibition may speed the apparent decay of the EPSCs in control conditions. However, this is not the only explanation, as application of APV partially reversed the effect of gabazine (rise time 1.34 ± 0.39 ms and decay: 11.2 ± 3.2 ms, n = 6). Most strikingly, APV mainly blocked the slow rising-phase of EPSCs and sustained build-up activity during paired electrical and optical stimulations (Suppl. Fig. 5), in agreement with a role of NMDA receptors in slow synaptic summation in granule cells and of inhibition in refining temporal transmission of information. These results validate the relevance of spatio-temporally patterned asynchronous optogenetic stimulations *in vitro* for the elucidation of brain microcircuits transfer functions.

## Discussion

Acousto-optic light steering has emerged as a powerful technology for functional optical imaging^56–60^. The fast switching times and random-access capabilities of AODs make them ideal for optical stimulation of neuronal activity. However, most former studies using AODs for optical stimulation involved the use of caged compounds^35,58,61^, which exhibit poor spatial specificity. Although more recent studies have demonstrated optogenetic stimulation with AODs, this has been done with relatively small beams (≈1.54 μm^2^)^62,63^ that limit addressing speed, spike precision and the latency, the latter reaching values up to to 5–10 ms^62^ which is an order of magnitude slower than what is currently attainable with fast opsins like Chronos^24,64,65^. Furthermore, high-NA beams do not improve the effective lateral precision in depth, as scattering spreads out a significant amount of the focal point energy. In this paper, we have boosted the switch time down to the sub-microsecond range by focusing the beam at the intermediate plane of a pair of crossed-oriented AODs. This configuration enabled us to extend the beam waist laterally at the focal plane of the microscope to match the size of our cellular targets (2–10 μm) and to mitigate the influence of scattering. This illumination pattern not only provided single-cell lateral precision but also a remarkably fast temporal (≤100 μs) stimulation precision. Stimulation precision was preserved during extended stimulation patterns, using an *ad-hoc* algorithm to generate illumination sequences of user-define targets that minimized stimulation crosstalk by spacing in time the illumination of nearby targets.

The speed of sequential AOD light addressing surpasses parallel light delivery methods based on amplitude and/or phase modulation. Liquid-crystal spatial light modulators exhibit refreshing rates up to a few kHz^66,67^ while digital micro-mirror devices of limited size may refresh up to tens of kHz^68–71^. Parallel illumination of several targets could increase stimulation rate but would inevitably lead to artificial synchrony, contrasting with the asynchronous activity patterns observed *in vivo*^72–74^. Even though it was not a limitation for our application, the excitation generated by our system lacks axial confinement and can deliver light precisely only within the first hundred microns of brain slice. Further spatial specificity could be achieved by using soma-targeted opsins^27,75,76^ or by using two-photon excitation that provides better optical sectioning and greater penetration depth. Furthermore, the latter option could be combined with temporal focusing^77^ for further improvement of the axial response offering the same spatial specificity as previously reported methods^21^ but much higher speeds. Moreover, a temporally focused low-NA Gaussian beam could be precisely positioned in a three-dimensional volume using a high-speed acoustic lens^59,78–81^ or adding chromatic dispersion^82^. Alternatively, one could perform beam shaping by feeding the AODs with chirped signals (or multiple acoustic frequencies)^81,83–85^ to create more power-efficient and axially confined light intensity patterns for optogenetic activation.

We used our AOD-based light delivery method to dissect the transfer function of the cerebellar granular layer, a monosynaptic excitatory feedforward circuit controlled by a single inhibitory interneuron. Based on the combinatorial nature of its synaptic connectivity, the granular layer has long been postulated to perform an optimal separation of its MF inputs through expansion recoding by granule cells and sparsening of granule cell activity by inhibition^86–88^. However experimental demonstration of these mechanisms at the network level is currently lacking. While injection of synthetic synaptic currents in single granules cells seemed to support linear input/output transmission of their combined 3 to 7 MF inputs^89,90^, recent experimental evidence suggests that convergent MF inputs from different modalities play dissimilar roles^55^ and that temporal summation of activity by the NMDA conductance combined with intrinsic granule cell non-linearities^53,54^ may produce complex frequency-dependent behavior. Network-level mechanisms engaging inhibition have been probed using electrical stimulation of the mossy fibers and demonstrated high-pass properties^91^ as well as non-linear lateral interactions^51^. However, electrical stimulations used in these studies synchronize artificially many MFs and evoke highly non-physiological synaptic inputs in which fast sub-millisecond AMPA components summate, thus bypassing subthreshold integration mechanisms. In this study we emulated low frequency asynchronous MF activity mimicking spontaneous proprioceptive-like discharge encountered *in vivo*, and paired it with juxta-threshold electrically evoked input, to avoid synchronizing many fibers. None of the two MF patterns presented alone evoked strong granule cell activity, seen here as EPSCs in Purkinje cells. However, when patterns were presented together, large EPSCs time-locked to the synchronous pattern of stimulation were observed in Purkinje cells. These results are similar to those obtained in the electrosensory lobe of the weakly electric fish *in vivo* where asynchronous sustained somatosensory inputs converge on granule cells together with time-locked efferent motor copy^92^. Our results are the first *in vitro* experimental demonstration of the hypothesis that convergence of mossy fibers generates combinatorial recoding by placing some salient and temporally-precise features (synchrony, onset, bursts) within the context of the ongoing sensory-motor stream. These results show how serial spatio-temporal light patterning techniques can be used to study neuronal integration and network behavior *in vitro* under *in vivo*-like regimes of activity.

*In vitro* patterned stimulation of network activity allows for pharmacological intervention and cell specific manipulations, which are not easily done *in vivo*. In the case of cerebellar processing, blocking synaptic inhibition dramatically increased the response recorded in Purkinje cells, confirming the crucial role played by inhibitory Golgi cells in the control of information transfer in the granular layer. Electrical stimulation cannot address the role of local interneurons, as they directly recruit local inhibitory axons. Previous hypotheses assigning a role for Golgi cells in gain control^86–88^ and time windowing^93^, as well as model predictions^94^, could thus be evaluated experimentally *in vitro* with our system. Asynchronous optical stimulation also allowed us to reveal the contribution of NMDA conductances on granule cell subthreshold integration, which is not easily assessed *in vivo*^95,96^, confirming previous results obtained *in vitro* with chemical network stimulations^54^. We propose that the non-linearity of granule cell responses results from a gain control process involving slow synaptic components (NMDA and high-affinity GABA), which keep granule cells at subthreshold levels during tonic optogenetic input, and a time windowing component, which preserves the temporal precision of responses to salient inputs. This configuration would implement efficient pattern recoding by granule cells while keeping the temporal precision of the transmitted information.

In summary, our method provides a framework to study brain microcircuits in a physiological context of activity while taking advantage of the analytical power of slice electrophysiology and imaging.

## Methods

All animal manipulations were made in accordance with guidelines of the Centre National de la Recherche Scientifique and protocols were approved by the French Ministry of Research and Higher Education under the protocol number 02235.02.

### Preparation of cerebellar slices

Sagittal slices (260–290 μm thick) were prepared from the cerebellum of transgenic mice where the expression of channel-rhodopsin2 was controlled either by the L7 promoter (L7-ChR2-YFP^48^) and therefore expressed only in cerebellar Purkinje cells (Fig. 3) or by Thy1 promoter (Thy1-ChR2-YFP; line18^49^) and expressed in the cerebellar mossy fibres (Figs 4–6). The animals (identified as positive through tail DNA PCR, using primers for thy1.2 and L7) of either sex, aged 2–4 months for L7-ChR2-YFP (P55–P127) and 1–3 months for Thy1-ChR2-YFP (P48–P84) were decapitated after deep anaesthesia induced with isofluorane (4% in medical oxygen). Cerebellum was quickly removed and rapidly dissected in an ice-cold bicarbonate-buffered solution (BBS) containing the following (in mM): 152 NaCl, 3.5 KCl, 1.25 NaH_2_PO_4_, 26 NaHCO_3_, 25 glucose, 1.6 CaCl_2_ and 1.5 MgCl_2_ supplemented with minocycline (50 nM) to inhibit the activation of microglia. Parasagittal slices of the cerebellar vermis were cut using a stainless steel blade (z deflection less than 0.5 µm) with an oscillating blade microtome (Campden Instruments) in an ice-cold gluconate-based cutting solution (GCS) containing (in mM): 130 K-gluconate, 15 KCl, 2 ethylene glycol-bis (2-aminoethyl ether)-N,N,N′,N′-tetraacetic acid (EGTA), 20 N-2-hydroxyethyl piperazine-N-2-ethanesulphonic acid (HEPES), 25 glucose, minocycline (50 nM), 0.05 D-(-)-2-amino-5-phosphonovaleric acid (D-APV) to prevent glutamate excitotoxicity and adjusted to pH 7.4 with NaOH. This solution was designed to mimic the intracellular medium and to limit the entry of calcium and other extracellular ions into cells, whose dendrites were cut during the slicing procedure^97^. Slices were then transiently immersed in a modified, mannitol-based solution (MCS) containing the following (in nM): 225 D-mannitol, 2.5 KCl, 1.25 NaH_2_PO_4_, 25 NaHCO_3_, 25 glucose, 0.8 CaCl_2_ and 8MgCl_2_ (34 °C, bubbled with 95% O_2_, 5% CO_2_) to help for progressive ions re-equilibration toward normal external concentrations. Finally, the slices were transferred to warm (34 °C) BBS, in which they were stored for up to 6–8 h.

### Electrophysiological recordings

For electrophysiological recordings slices were moved to the recording chamber mounted on an upright Olympus BX51WI modular microscope equipped with a x40 water-immersion objective (LUMPlanFL/IR, 0.8NA) and continuously perfused with gassed BBS (perfusion was regulated by a peristaltic pump with a flow rate ~3.5 ml/min; 33 °C). Neurons were visualized for patch-clamp recordings using a combination of gradient contrast and online video contrast enhancement (CoolSNAP cf, Photometrics and MetaVue, Roper Scientific). Patch pipettes (3.3–3.6 MΩ for Golgi cells and 2.5–3.0 MΩ for Purkinje cells) were pulled from borosilicate glass capillaries (0.15 mm diameter, Hilgenberg) with a vertical puller (David Kopf Instruments) and coated with dental wax. Recordings were obtained using an Axopatch 200B and MultiClamp 700B amplifier (Molecular Devices) and converted by a Digidata 1320 A interface (Molecular Devices). Signal was low-pass filtered at 6 kHz and acquired at 20 kHz. Golgi cells (GoCs) could be unambiguously differentiated from other cells in the granular layer by the size of their soma (10–25 µm), Purkinje cells (PCs) were easily distinguished from other cell types in the cerebellar cortex by their big soma and the position between granular and molecular layer; further both cells have characteristic bi-exponential capacitive current^98^.

For whole-cell recordings of Golgi and Purkinje cells pipettes were filled with a physiological intracellular solution containing (in mM): 150 KMeSO_4_, 6NaCl, 10 HEPES, 0.03 EGTA, 1 MgCl_2_, 4 ATP-Mg and 0.4 GTP-Na with pH adjusted to 7.35 by 1 M KOH. During whole-cell recordings the holding potential of voltage clamped GoC and PC was -60mV (correcting for junction potential). In several experiments cells were filled with the morphological dye Alexa 594 (15 μM; Invitrogen, Carlsbad, CA) through the whole-cell patch pipette.

Experiments with currents evoked directly in PC in L7-ChR2 mice were performed with the presence of blockers of synaptic activity (TTX 200 nM (Sigma); SR 95531 5 µM (gabazine, Abcam); NBQX 2 µM (Abcam)) to prevent any contamination of the current by the synaptic events. Stimulation was every 10 s.

Optical stimulation of MFs performed in Thy1-ChR2-YFP animals and recordings in GoC experiments were performed in the presence of blockers of inhibition (SR 95531(gabazine) 5 µM and strychnine 1 µM). 200 nM TTX was added during the recordings to verify that the EPSCs evoked by single spot laser stimulation of MFs- are action potential mediated. Stimulation was every 1 s.

Spatial resolution experiments: in the presence of blockers of synaptic inhibition (SR 95531 (gabazine) 5 µM and strychnine 1 µM) and 15 µM 4-aminopyridine (4AP). Each point was stimulated for 1 ms every 1 s.

Starting from the emulation of MF sensory-motor activity experiments (Figs 5 and 6), we have noticed that the density of ChR2 expression may not be high enough in the Thy1-ChR2-YFP transgenic mice to evoke robust MF activity. Low concentrations (10 µM) of 4-aminopyridine (4-AP), a potassium channel blocker was used to increase the excitability of axons and greatly improved our capacity to evoke action potentials in MFs.

### Data analysis

Electrophysiological data were analysed in Clampfit 10.4 (Molecular devices); statistical analysis was performed using RStudio. 10 sweeps were averaged for light evoked currents recorded in Purkinje cell; 80–100 sweeps were averaged for the mossy fibre single spot stimulation.

Averages of pharmacologically modulated traces were done only after ~6 minutes from the beginning of the perfusion of the drug into the recording chamber to provide the time for the steady state effect. Values are given as an average ± s.d., unless stated differently.

### General system design

This paper describes an optogenetic stimulation system based on acousto-optic steering of low-NA Gaussian beams. To obtain such beams, we focused a laser beam at the medial plane of a dual-axis AOD scanner and used a variable beam expander to control the beam diameter (2*W*_1_) at such plane. This enabled us to balance illuminated volume and switching time, which is linearly proportional to the beam expander magnification: ${t}_{switching}\approx {B}_{diameter}{M}_{BE}/{v}_{{p}_{Te{O}_{2}}}$. The back focal plane of the objective was optically conjugated to the AOD’s plane by a 4f-system that, together with the maximum deflection angle and the objective, determine the size of the FOV: $FOV\approx {\rm{\Delta }}{\theta }_{{D}_{max}}\frac{{f}_{1}}{{f}_{2}}{f}_{obj}$. Choosing the proper magnification for the beam expander (2–5x) enabled us to adjust the spot diameter (2*W*_2_) to the size of our targeted structure within a range from 2–5 µm (10 µm removing the beam expander).

### Optimized spatiotemporal light patterns

We developed an algorithm for spatio-temporal pattern generation of illumination spots that maximizes the illuminated surface and assure constant overall power over time and space within our targeted area.

First, the algorithm creates a mesh of points uniformly distributed within the targeted area. This mesh, in the form of a binary image, sets both the accessible points and the minimum distance between illumination points. Then the first point in the sequence is randomly chosen and the rest are selected according to a decision map (or time-weight distance function) that assigns a score to all potential locations for the n-th element of a sequence. This score is given by the equation below

2 $${S}_{n}(x,y)=\,{\rm{\min }}(W(n)\times dist(Map(x,y,n)))$$

where *W*(*n*) is the time-weight factor, *dist* represents the geometrical distance function and *Map* is a binary map encoding the position of the already accessed points in the sequence. Additionally, the distance function is mirrored to avoid a bias towards the edge on the decision map. Lastly, the grid point with the highest score is selected as the next point in the sequence and the process repeated until the end of it.

### Diffraction efficiency

The coupled-wave equations that describe the anisotropic Bragg diffraction for non-collinear light-sound propagation waves in a birefringent medium have been previously described^99^ and solved^100,101^. From these equations, we can derive the diffraction efficiency (*DE*) (defined as the intensity ration between the diffracted intensity (*I*_*d*_) and the intensity in the zero-order (*I*_0_)):

3 $$DE=\frac{{{\rm{I}}}_{{\rm{d}}}}{{{\rm{I}}}_{0}}={\sin }^{2}({K}_{1}\sqrt{{P}_{a}})$$

where *K*_1_ is a constant that depends on the geometry of the crystal and the illumination and *P*_*a*_ is the acoustic power. The diffraction efficiency of our AODs is characterize on (Suppl. Fig. 1a).

### Bandshape correction

Tangential phase matching is one of the most suitable interaction geometries for AODs because it allows broader acoustic frequency ranges satisfying the phase matching condition. One of the first practical AOD implementations reported used this configuration in combination with the extremely high diffraction efficiency of TeO_2_ although its bandwidth was relatively narrow^102^. Later implementations enable extended bandwidths, but suffered from a midband degeneracy or “dip”^103^. This effect, due to rediffraction, was partially suppressed using a slightly tilted direction for the acoustic wave, an alternative geometry based on the one proposed by Uchida *et al*.^31^. This type of interaction geometries is still most frequently used^104^ as it provides a broad yet rippled bandshape (in our device reaching up to 25.6% variations, Suppl. Fig. 1b).

We modulated the acoustic power according to the bandshape variations^35^, in order to deliver equal laser power regardless of the deflection angle. This resulted in a much uniform light delivery on the FOV; 2.8% intensity variation after one iteration of the algorithm (Suppl. Fig. 1c) and 2.6% on the second iteration (Suppl. Fig. 1d).

### ChR2 current recruitment model

The interaction between protein and light can be modeled by a Poisson distribution with rate $\lambda =\frac{RE(P)}{R{E}_{0}}$, where *RE*(*P*) is the radiant exposure for power P and *RE*_0_ is the radiant exposure when the light-protein interaction probability drops by a factor of *e*^−1^. Thus, the probability that the channel is opened by any of the arriving photons is given by

$$P(\lambda ,X\ge 1)=1-\exp (-\,\lambda )$$

Using this probability we can model ChR2 current recruitment (*C*_ChR2_) as follows:

4 $${{\rm{C}}}_{{\rm{ChR}}2}={\int }_{0}^{2{\rm{\pi }}}{\int }_{0}^{\infty }\,{{\rm{d}}}_{{\rm{ChR}}2}(1-\exp (-\,\frac{{\rm{RE}}}{{{\rm{RE}}}_{0}})){\rm{r}}\,{\rm{dr}}\,d{\rm{\theta }}$$

where *r* is the polar coordinate, *d*_*ChR*2_ is the ChR2 current density. We define the radiant exposure distribution as

5 $$RE(r)=\frac{P\tau }{2\pi {\sigma }_{r}^{2}}\exp (-\,\frac{1}{2}{(\frac{r}{{\sigma }_{r}})}^{2})$$

where *P*, *τ* and *σ*_*r*_ are the power, pulse duration and the spatial standard deviation of the light distribution respectively.

Substituting Eq. 5 in Eq. 4 we obtain

6 $$\begin{array}{ccc}{C}_{ChR2} & = & {\int }_{0}^{2\pi }{\int }_{0}^{\infty }{d}_{ChR2}(1-\exp (-\,\frac{1}{{I}_{0}}\frac{P}{2\pi {\sigma }_{r}^{2}}\exp (-\,\frac{1}{2}{(\frac{r}{{\sigma }_{r}})}^{2})))r\,drd\,\theta \\  & = & \mathop{\underbrace{2\pi {d}_{ChR2}}}\limits_{a}{\int }_{0}^{\infty }(1-\exp (-\,\mathop{\underbrace{\frac{1}{{I}_{0}}\frac{P}{2\pi {\sigma }_{r}^{2}}}}\limits_{b}\exp (-\,\mathop{\underbrace{\frac{1}{2{\sigma }_{r}^{2}}}}\limits_{c}{r}^{2})))r\,dr\end{array}$$

which can be rewritten in a more compact form by grouping the parameters

7 $${C}_{ChR2}=a{\int }_{0}^{\infty }(1-\exp (-\,b\,\exp (-c{r}^{2})))r\,dr$$

Using the following change of variables

8 $$\{\begin{array}{ccc}t=b\,\exp (\,-\,c{r}^{2}) & \Rightarrow  & r=\pm \sqrt{-\frac{1}{c}\,\mathrm{ln}(\frac{t}{b})}\\ dt=-\,2bcr\,\exp (\,-\,c{r}^{2})dr & \Rightarrow  & dr=\frac{dt}{-2c\sqrt{-\frac{1}{c}\,\mathrm{ln}(\frac{t}{b})}t}\end{array}$$

on Eq. 7 we obtain

9 $$\begin{array}{ccc}{C}_{ChR2} & = & a{\int }_{0}^{\infty }(1-\exp (-b\,\exp (-c{r}^{2})))r\,dr\\  & = & a{\int }_{b}^{0}\frac{(1-\exp (-t))\sqrt{-\frac{1}{c}\,\mathrm{ln}(\frac{t}{b})}}{-2c\sqrt{-\frac{1}{c}\,\mathrm{ln}(\frac{t}{b})}t}dt\\  & = & \frac{a}{2c}{\int }_{0}^{b}\frac{1-\exp (-t)}{t}dt\end{array}$$

which has the following known solution^105^:

10 $${\int }_{0}^{z}\frac{1-\exp (-\,t)}{t}dt={E}_{1}(z)+\,\mathrm{ln}(z)+\gamma $$

where *E*_1_(*z*) is the exponential integral given by ${E}_{1}(z)=-\,\gamma -\,\mathrm{ln}(z)-\sum _{n=1}^{\infty }\frac{{(-1)}^{n}{z}^{n}}{nn!}$ and *γ* is the Euler’s constant. Thus, we can rewrite Eq. 9 as

11 $$\begin{array}{ccc}{C}_{ChR2} & = & \frac{a}{2c}{\int }_{0}^{b}\,\frac{1-\exp (-\,t)}{t}dt\\  & = & \frac{a}{2c}[{E}_{1}(b)+\,\mathrm{ln}(b)+\gamma ]\\  & = & \frac{a}{2c}\sum _{n=1}^{\infty }\,\frac{{(-1)}^{n+1}{b}^{n}}{nn!}\end{array}$$

Finally a solution to Eq. 4 can be expressed as follows

12 $${C}_{ChR2}=2\pi {\sigma }_{r}^{2}{d}_{ChR2}\sum _{n=1}^{\infty }\,\frac{{(-1)}^{n+1}{(\frac{{\boldsymbol{P}}\tau }{2\pi {\sigma }_{r}^{2}}/R{E}_{0})}^{n}}{nn!}$$

To account for scattered light we added an additional parameter *α*_*s*_ that gives rise to an increase in ChR-2 current proportional to the light power:

13 $${C}_{ChR2}=2\pi {\sigma }_{r}^{2}{d}_{ChR2}\sum _{n=1}^{\infty }\,\frac{{(-1)}^{n+1}{(\frac{{\boldsymbol{P}}\tau }{2\pi {\sigma }_{r}^{2}}/R{E}_{0})}^{n}}{nn!}+{\alpha }_{s}{\boldsymbol{P}}\tau \,$$

## Electronic supplementary material

Dataset 1

Supplementary Video 1: Raster scanning with 10 µm spacing.

Supplementary Video 2: Random-access scanning with 0.25 µm minimum spacing.

Supplementary Video 3: Random-access scanning with 10 µm minimum spacing.

Supplementary Video 4: Random-access scanning with 10 µm minimum spacing and no-revisiting condition.

Supplementary Video 5: Optimized random-access scanning with 10 µm minimum spacing.

Supplementary Video 6: Optimized random-access scanning for arbitrary patterns.

## Data Availability

The data and computer code that support the findings of this study are available from the corresponding author upon reasonable request.
