# Supplementary material for: Optogenetic stimulation of complex spatio-temporal activity patterns by acousto-optic light steering probes cerebellar granular layer integrative properties
Source: Sci Rep. 2018 Sep 13;8:13768. doi: 10.1038/s41598-018-32017-w (PMC6137064; doi:10.1038/s41598-018-32017-w)
Supplement: Supplementary file 1 — Dataset 1 [file 41598_2018_32017_MOESM1_ESM.docx]

Optogenetic stimulation of complex spatio-temporal activity patterns by acousto-optic light steering probes cerebellar granular layer integrative properties

Oscar Hernandez ^1,2,3,4,*,†^, Katarzyna Pietrajtis ^1,2,3,^*, Benjamin Mathieu ^1,2,3^ and Stéphane Dieudonné ^1,2,3^

**
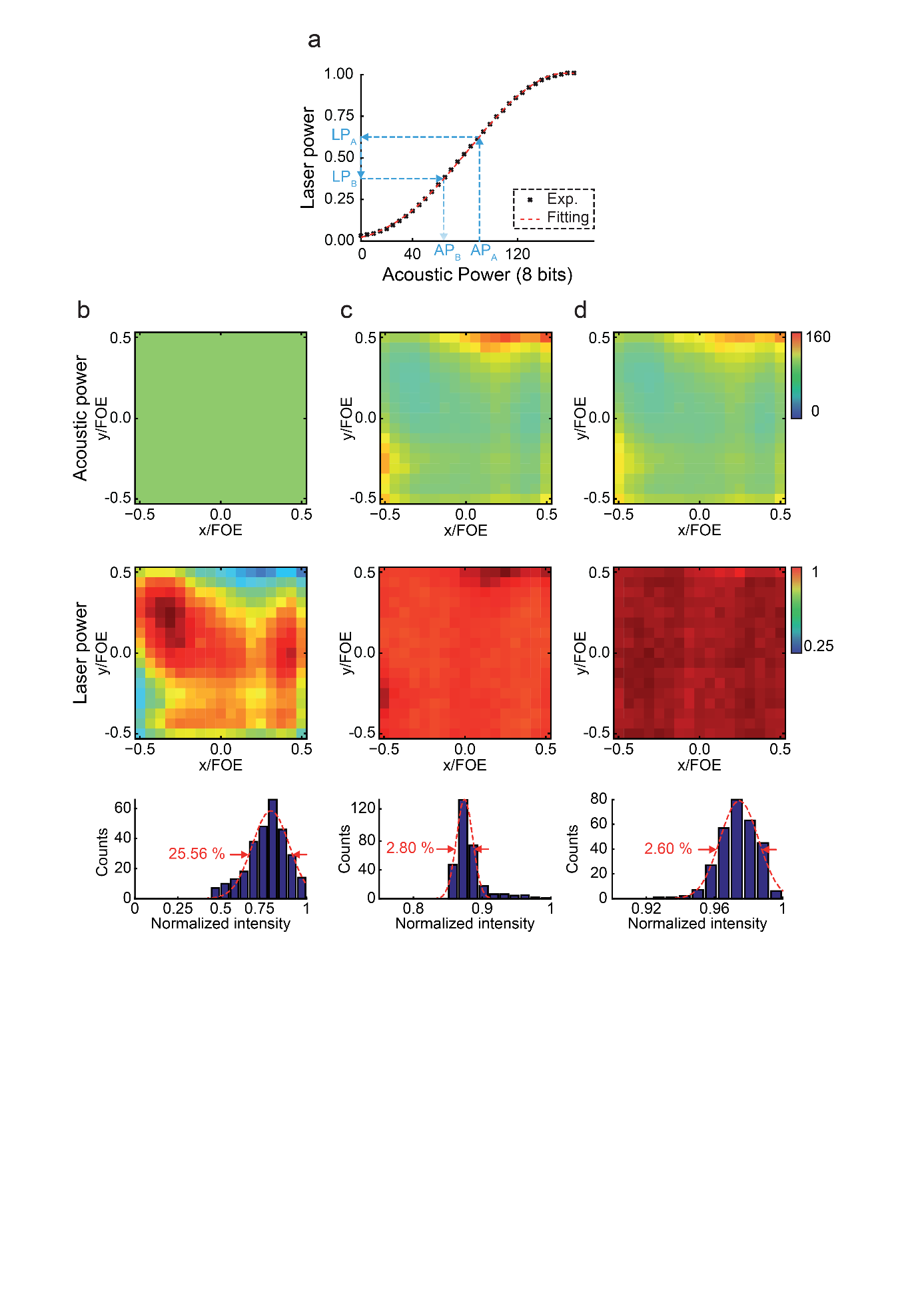
**

**Supplementary Figure 1.** Laser power equalization. **a,** Characterization of the output laser power for increasing values of acoustic power. The experimental data points (black crosses) follow an expected sinusoidal behavior (see Materials and Methods). A deflection-angle efficiency dependency translates into a multiplicative factor over the output laser power. Because of this, the output laser power differs (LP_A,_ LP_B_) for distinct deflection angles but same acoustic power (AP_A_) but can be compensated interpolating and obtaining a corrected acoustic power (AP_B_). **b-d**, In the different columns we show the acoustic power, laser power distribution and laser power histogram obtain after illuminating of the entire field of excitation using constant acoustic power (first column), corrected acoustic power interpolating once on **(a)** (middle column) and performing a second interpolation on the corrected acoustic power (right column).


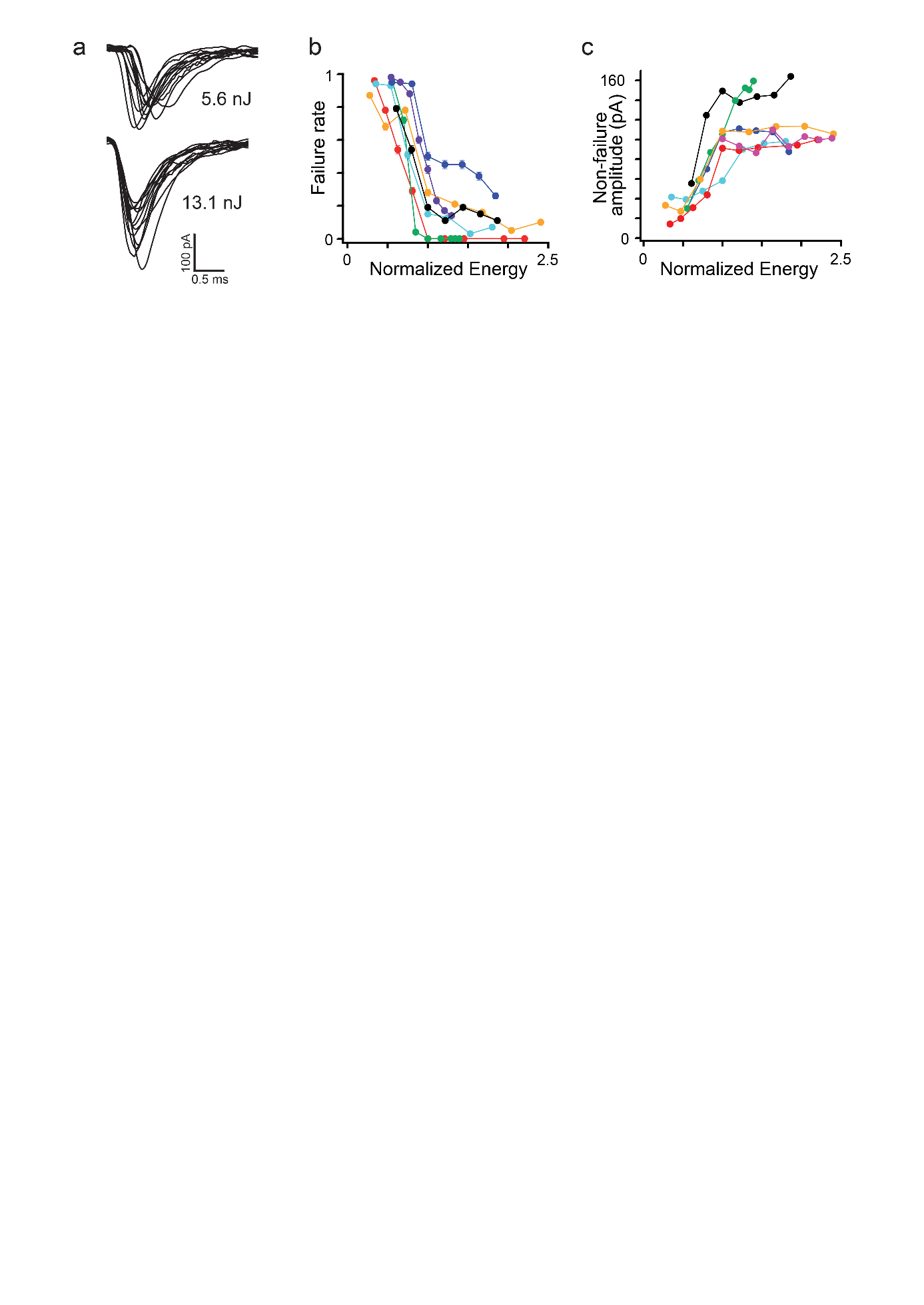


**Supplementary Figure 2.** Biophysics of single mossy fiber stimulation. **a.** Examples of light evoked EPSCs at two different energies close to the ChR2 saturation level. Note smaller jitter of the evoked events at higher energy. **b.** Graph presenting failure rate as a function of energy. Energy was normalized to the half activation value, extracted from sigmoidal fit applied to the recruitment curves. **c.** Graph showing non-failure amplitude of light evoked EPSCs as a function of energy during recruitment curve. Energy was normalized as in **(b)**.


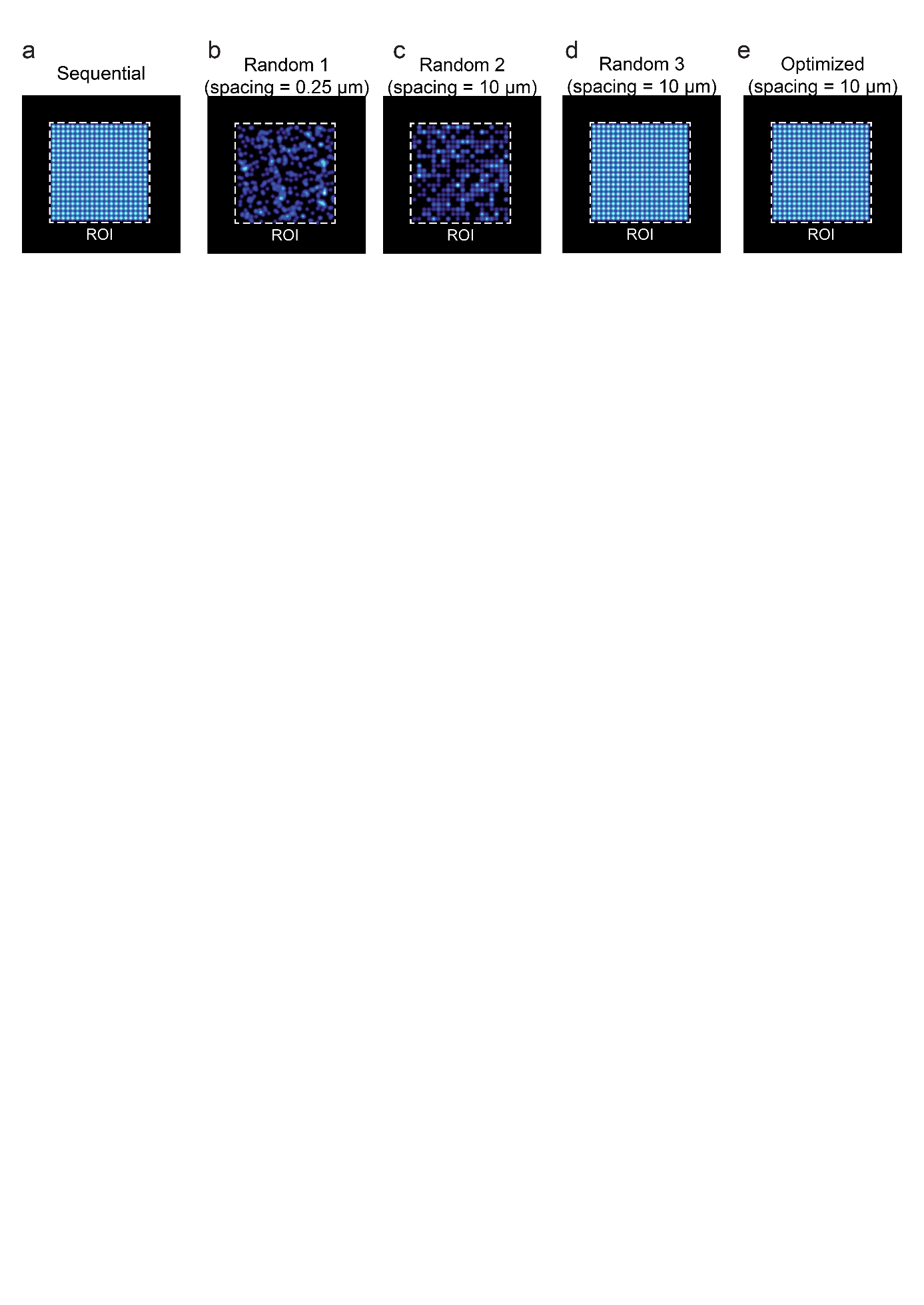


**Supplementary Figure 3.** Random-access laser scanning and light coverage. Homogeneous light distribution in time and space within an arbitrary region of interest (ROI) was achieved using distinct sequence generation algorithms. The panels show the maximum projection of temporal sequences of 400 spots whose location and timing was determined using: raster scanning with 10 µm spacing between adjacent points **(a, Suppl. Video 1)**, random-access scanning with minimum spacing of 0.25 µm between adjacent points **(b, Suppl. Video 2)**, random-access scanning with 10 µm minimum spacing **(c, Suppl. Video 3),** random-access scanning with 10 µm minimum spacing and no-revisiting condition **(d, Suppl. Video 4)** and our optimized sequence generation algorithm using 10 µm minimum spacing **(e, Materials and Methods, Suppl. Video 5-6)**.

**
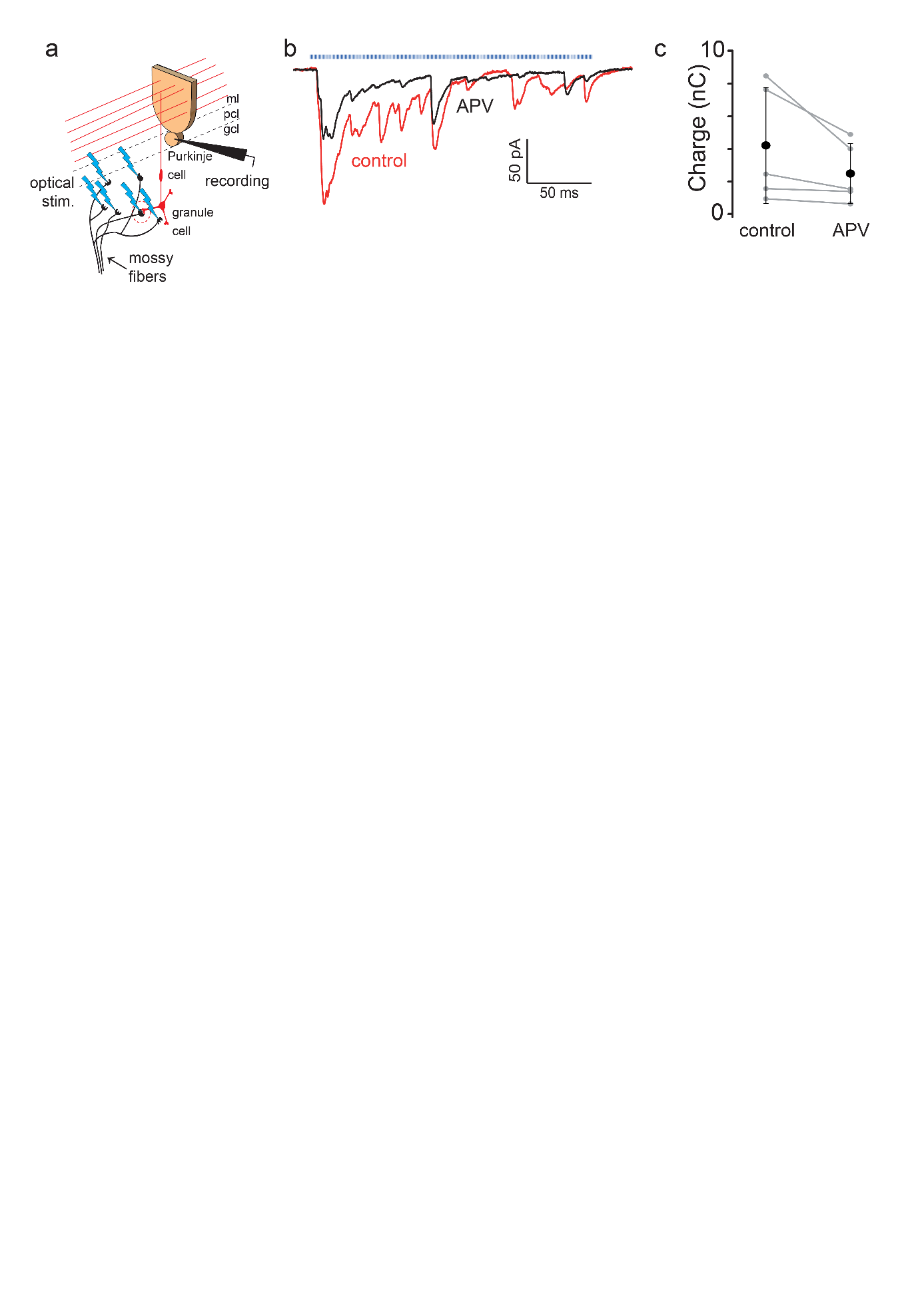
**

**Supplementary Figure 4.** Light activation of mossy fibers is strong enough to be relayed on granule cells. **a.** Schematic drawing showing experimental configuration. GCL output was monitored by recordings from Purkinje cells in Thy1-ChR2-YFP and asynchronous pattern of optical stimulation of mossy fibers was applied (blue lightning). **b.** Mossy fiber stimulation was strong enough to evoke disynaptic response of granule cells. Examples of traces recorded during the experiments presented in a; red trace shows response recorded in Purkinje cells, transmitted from light evoked mossy fiber inputs in control conditions and after application of APV (in black). **c.** Charge carried during light mossy fiber stimulation recorded in Purkinje cells (n=5) in control conditions and with application of APV.


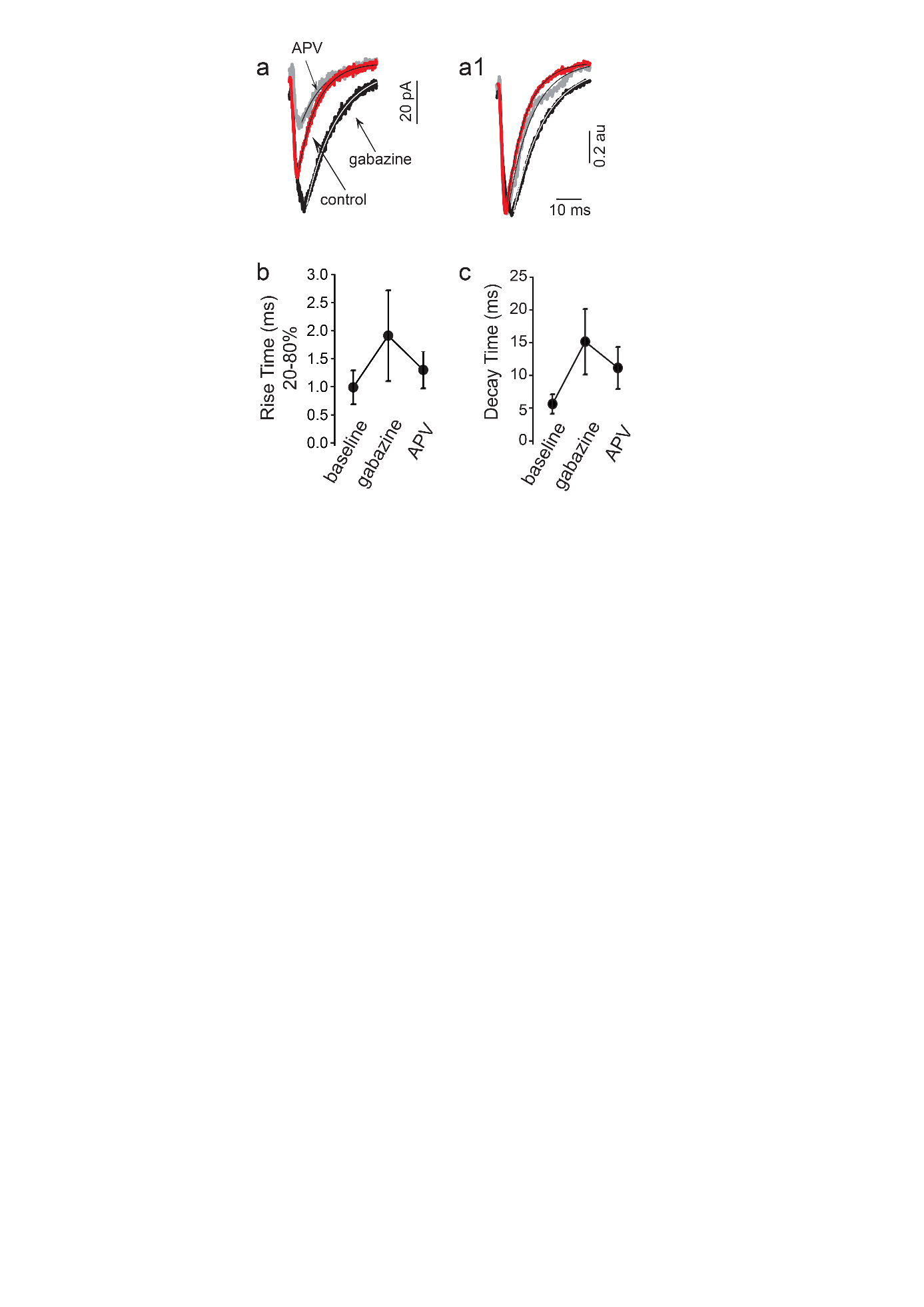


**Supplementary Figure 5.** Kinetics of the potentiated events changes in pharmacological conditions. **a.** Average EPSCs recorded in the Purkinje cell upon paired electrical and optical stimulation (see Fig. 6) in control conditions (red trace), after gabazine (5 µM, black trace) and APV (50 µM, grey trace) application. **a1**. Peak-scaled and aligned on the rise time averaged EPSCs (same as in a) showing that after gabazine application (black trace) the decay time of the event is slower than in control conditions (red). After APV application (grey trace) decay time is partially restored. Black lines (and white line in black trace) indicate exponential fits to the decay phase (ctrl: 5.7 ± 1.5 ms; gabazine: 15.2 ± 5 ms; APV: 11.2 ± 3.2 ms) **b.** Rise time of the events is similarly affected by the pharmacological conditions. Summary of rise times (20-80%) in control condition (1.0 ± 0.28 ms), after application of gabazine (1.95 ± 0.73) and APV (1.34 ± 0.39 ms; n=6). **c.** Summary of the decay time constants (ms) in three pharmacological conditions (n= 6 cells). In b and c, black dots represents averages and error bars are ± s.d.

**Supplementary Video 1.** Raster scanning with 10 µm spacing. Spatiotemporal light sequence of 400 illumination points generated using random-access raster scanning with 10 µm spacing between adjacent points. The illumination pattern homogeneously covers the region of interest (ROI) (**Suppl. Fig. 3.a**). However, ever point new point in the sequence is adjacent to the previous point.

**Supplementary Video 2.** Random-access scanning with 0.25 µm minimum spacing. Spatiotemporal light sequence of 400 illumination points generated random-access scanning with minimum spacing of 0.25 µm between adjacent points. The illumination pattern partially covers the ROI (**Suppl. Figure 3.b**). However, consecutive points in the sequence occupy overlapping positions in space or even revisit the same position. These points are highlighted in the video with a dashed blue line rectangle.

**Supplementary Video 3.** Random-access scanning with 10 µm minimum spacing. Spatiotemporal light sequence of 400 illumination points generated random-access scanning with 10 µm minimum spacing. The illumination pattern partially covers the ROI (**Suppl. Fig. 3.c**). However, consecutive points in the sequence occupy close positions in space or even revisit the same position. These points are highlighted in the video with a dashed blue line rectangle.

**Supplementary Video 4.** Random-access scanning with 10 µm minimum spacing and no-revisiting condition. Spatiotemporal light sequence of 400 illumination points generated random-access scanning with 10 µm minimum spacing between adjacent points. The illumination pattern partially covers the ROI (**Suppl. Fig. 3.d**). However, consecutive points in the sequence occupy close positions in space. These points are highlighted in the video with a dashed blue line rectangle.

**Supplementary Video 5.** Optimized random-access scanning with 10 µm minimum spacing. Spatiotemporal light sequence of 400 illumination points generated random-access scanning with minimum spacing of 10 µm between adjacent points. The illumination pattern partially covers the ROI (**Suppl. Fig. 3.e**). However, consecutive points in the sequence occupy close positions in space. These points are highlighted in the video with a dashed blue line rectangle.

**Supplementary Video 6.** Optimized random-access scanning for arbitrary patterns. Experimental fluorescence intensity images recorded at a fixed exposure time exposure and illumination time of 1 ms. Averaging sets of 10, 50 and 100 images, we can observe how the spatiotemporal light sequence of illumination forms a user-defined pattern depicting the letters ENS. However, the illumination points are spaced 10 µm and the different points are accessed according to the optimized random-access scanning algorithm previously described (**Suppl. Fig. 3.e** and **Suppl. Video 5**).
